# PTSD in prison settings: A systematic review and meta-analysis of comorbid mental disorders and problematic behaviours

**DOI:** 10.1371/journal.pone.0222407

**Published:** 2019-09-26

**Authors:** Emma Facer-Irwin, Nigel J. Blackwood, Annie Bird, Hannah Dickson, Daniel McGlade, Filipa Alves-Costa, Deirdre MacManus

**Affiliations:** Department of Forensic and Neurodevelopmental Sciences, Institute of Psychiatry, Psychology and Neuroscience, King’s College London, United Kingdom; Harvard University, UNITED STATES

## Abstract

**Purpose:**

Prevalence rates of PTSD are higher in the prison population than in the community. We sought to systematically review the extent to which this disorder is associated with other mental health disorders and problematic suicidal or aggressive behaviours in the prison population.

**Methods:**

Studies reporting a relationship between PTSD and comorbid mental disorders and/or problematic behaviours in imprisoned adolescent and adult populations were identified from four bibliographic indexes. Primary studies involving clinical interviews, validated instruments leading to DSM or ICD diagnoses, or validated self-report questionnaires such as the PTSD checklist were included. Random-effects meta-analysis was conducted where possible. Preferred Reporting Items for Systematic Reviews and Meta-Analyses (PRISMA) guidelines were followed.

**Results:**

This review identified 36 studies, with a combined sample of 9594 participants, (6478 male and 2847 female prisoners) from 11 countries. Thirty-four of the identified studies employed a cross-sectional design. We identified significant associations between PTSD and comorbid mental disorders including depression (OR = 3.4, 95% confidence interval (CI): 2.3–4.9), anxiety (OR = 2.9, 95% confidence interval (CI): 1.8–4.7) and substance use (OR = 1.9, 95% confidence interval (CI): 1.5–2.4). We also identified significant associations between PTSD and suicidality (OR = 3, 95% confidence interval (CI): 2.4–3.8) and aggressive behaviours (this latter finding was not subject to meta-analysis). Significant methodological heterogeneity was identified between studies.

**Conclusions:**

High rates of psychiatric comorbidity among prisoners with PTSD, and links to suicidal behaviour, self-harm and aggressive behaviour, provide further support for the need for trauma-informed treatment approaches in prisons. However, significant gaps in the current evidence were apparent. In particular, a lack of large, longitudinal studies meant that the temporal relationships between PTSD and relevant outcomes cannot currently be determined.

## Introduction

High levels of lifetime traumatic exposures have been reported in studies of prison populations [1, 2]. A recent international meta-analysis confirmed that the prevalence of PTSD in prison populations, like other mental disorders, is higher than in community populations, with a pooled point prevalence of 6% in male prisoners and 21% in female prisoners [3].

High rates of other mental disorders, and problematic behaviours such as suicidal and aggressive behaviours have also been extensively documented in prison populations [4–6]. The relationship between PTSD and these outcomes is poorly understood and this may be perpetuating under-diagnosis and under-treatment of PTSD in prisons [7]. Community and military population studies have suggested that PTSD is a disorder which is highly comorbid with other mental health disorders [8], such as depression [9] and substance misuse [10, 11]. PTSD has also been linked to suicidality [12], self-harm [13], criminality [14], violence and aggressive behaviour [15–18] in community, clinical and military population studies.

In the present study, we examined the associations between PTSD and comorbid mental disorders or problematic behaviours in 9594 imprisoned individuals. To the authors’ knowledge, this study is the first meta-analysis that examines such associations in the adolescent and adult prison population.

## Methods

This systematic review protocol was pre-registered in PROSPERO (CRD42017068958) and PRSIMA guidelines were followed [19].

### Search strategies

**Fig 1:**
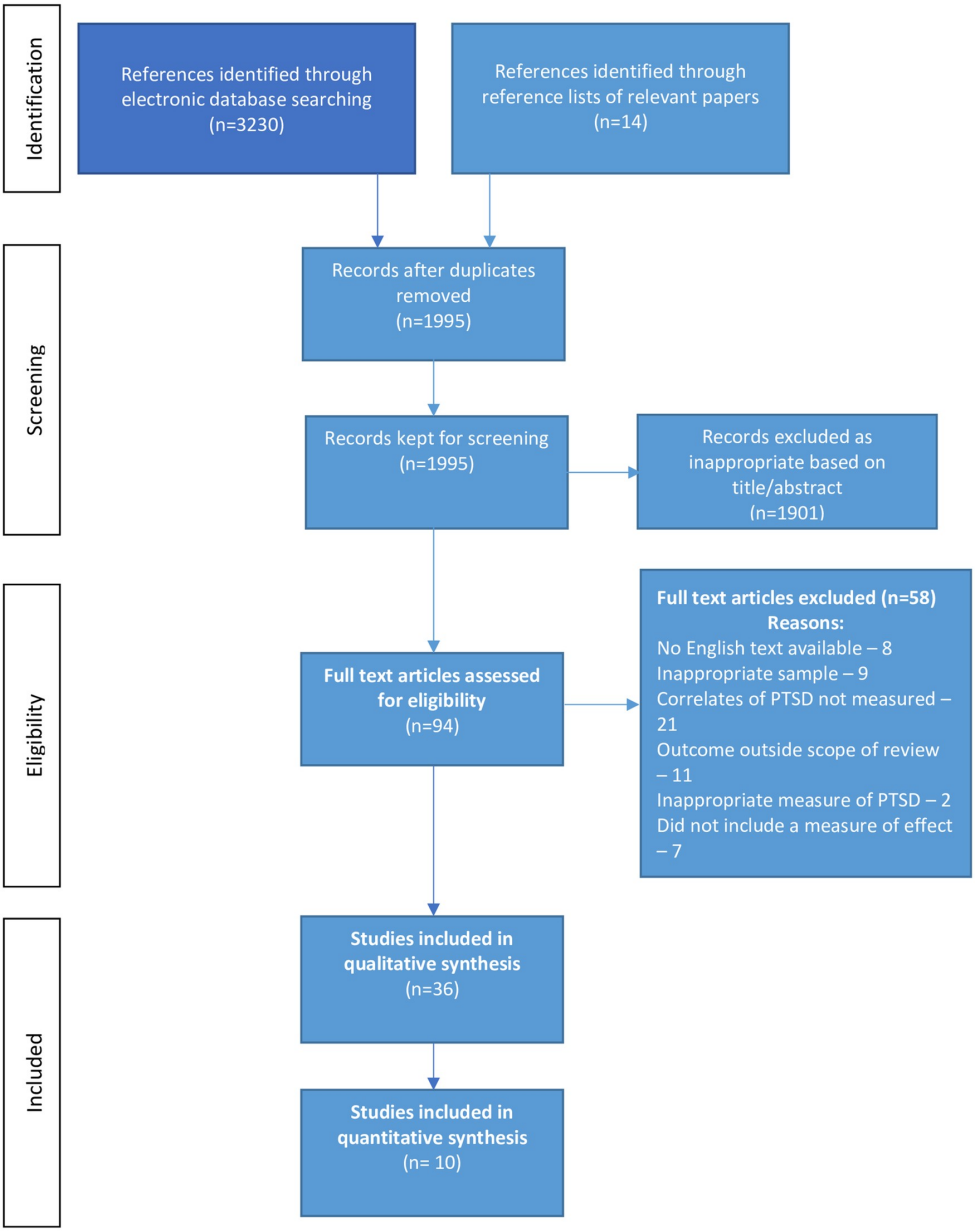
Systematic literature search flow.

We conducted a systematic search of the PTSD literature in prison populations, last updated on February 9^th^, 2019. The search included four online databases (Embase, MEDLINE, PsycINFO, Web of Science) and reference lists of identified papers and relevant systematic reviews [3]. For the online database searches, we used an identical combined strategy of free-text strings and subject headings (see S1 Text). Fig 1 describes the study selection process.

### Inclusion and exclusion criteria

We identified studies in which associations between PTSD and relevant correlates were reported. The following inclusion criteria were applied: 1) youth or adult prison samples (where the proportion of under 18 year olds represented less than 10% of the entire sample, the study was considered representative of adult prisoners); 2) probable PTSD diagnoses were established with validated diagnostic instruments such as the Structured Clinical Interview for DSM-5 [20] or validated self-report questionnaires such as the PTSD checklist [21]; 3) the relationship between PTSD and at least one mental health comorbidity, or problematic behaviour was examined; and 4) studies of male, female or mixed samples (in cases where the proportion of females represented less than 10% of the entire sample, the study was considered representative of male sex).

Studies meeting the following criteria were excluded: 1) investigations in prisoners of war, or other criminal justice settings e.g. probation, court; 2) no measure of association or effect; or 3) outcomes outside the scope of this review (i.e. physical health problems).

Titles and abstracts were screened against the inclusion and exclusion criteria. The remaining full texts of potentially eligible studies were then evaluated. Quality appraisals of included studies were performed by two independent postgraduate-level reviewers (EFI, FAC) using a checklist adapted from validated tools (see S1 Table; [22–25]). The total possible quality rating ranges from 0 to 42 points. Samples with a score of 32 and above were considered high quality; those with scores between 22 and 32 were considered medium quality; and those with scores of 21 or below were considered low quality. Disagreements between these two reviewers were resolved by consensus with a third senior reviewer (DM).

### Statistical analyses

While use of meta-analyses was precluded for most relationships due to an insufficient number of studies, meta-analyses were conducted for three comorbid mental disorders—Depression, Generalised Anxiety Disorder, and Substance Use Disorder and one behavioural association, namely suicidality. Meta-analyses were conducted with Stata version 15.1 using pooled random effects odds ratios.

The significance and the magnitude of heterogeneity across studies were calculated using the Q statistic and I^2^ statistic; significantly high levels of heterogeneity were indicated for Anxiety and Depression, but not for SUD or suicidality. Subgroup analyses were performed to examine differences according to gender, age, and timing of PTSD diagnosis (current or lifetime). For the purposes of analysis, “Current” timing included studies measuring PTSD at one month, 6 month and 12-month diagnostic periods. One study of incarcerated youth [26] did not provide a breakdown by gender and was therefore excluded from the gender subgroup analyses. One study [27] measured two diagnoses relating to SUD (substance abuse and substance dependence), and so both of these were included in analysis. Measures of suicidality included in the meta-analysis included lifetime measures of suicidal behaviour or ideation (n = 5) and current suicide risk (n = 2). There were insufficient studies to provide adequate statistical power for meta-regression, precluding further examination of effect size moderators.

## Results

### Key features of included studies

The 36 studies [26–61] reported on a combined sample of 9594 participants, 6478 male and 2847 female prisoners. Stratified by age, identified studies reported on a combined sample of 4139 incarcerated young offenders (age range 10–19) (M = 2766; F = 1324) and 5455 adult prisoners (M = 3712; F = 1523). The key characteristics of included studies are summarised in Table 1.

**Table 1:** Key features of included studies.

Study Design	Number of studies
Cross sectional	34
Cohort	2
Gender	
Males/boys	21*
Females/girls	19*
Males/females together- unable to get separate data	7
Age	
Youth samples	14
Adult samples	22
Timing of PTSD	
Point prevalence (i.e. past month, week)	24
Past year	4
Lifetime	9
Unclear	1
PTSD measure	
Diagnostic interview	18
Validated self-report tool	18
Problems examined*	
Comorbid mental disorders	21
Substance and Alcohol Misuse	16
Aggression and violence	9
Suicidality and Self-Harm	12
Offending behaviour (e.g. recidivism)	5
Quality appraisal score	
Low	6
Medium	26
High	4

*as categories (Gender and Associated problems) are not mutually exclusive, totals may exceed 36

Most studies were conducted in high income countries, with the majority (n = 21) conducted in the USA, four studies conducted in Europe [31, 35, 39, 55], and five studies (three reporting on the same sample) from the UK [47, 48, 50, 56, 58]. 22 studies reported on adult prisoners [39–60], while 14 reported on incarcerated youth [26–38, 61].

Half of the studies utilised a validated structured diagnostic interview to assess participants for PTSD (n = 18) [26–28, 30, 32, 34, 35, 38, 41–43, 46, 49, 51, 54, 55, 57, 60], with the remainder using validated self-report screening questionnaires. Although the reported prevalence of PTSD was typically higher among studies which used a questionnaire with a cut-off range, magnitudes of associations did not appear to differ substantially between such studies and those which used a diagnostic interview, although this could not be examined quantitatively due to an insufficient number of studies.

There was some heterogeneity between studies in the time-period within which PTSD was measured. The majority (n = 22) assessed current PTSD (i.e. past month, past week), but several studies [30, 34, 38, 41, 42, 51, 55, 57] measured lifetime prevalence and four measured 6-or 12-month prevalence [28, 43, 46, 49]. The overwhelming majority (94%) were cross-sectional studies, with only two studies [38, 51] employing any prospective/ longitudinal element. 18 studies measured associations between PTSD and other mental disorders, and 25 studies examined at least one behavioural problem. Of the 36 studies included in this review, 11% were considered high quality, 72% medium-quality and 17% low quality. Many studies [26, 27, 31–37, 39, 40, 43, 45–57, 61] reported associations between PTSD and other mental disorders or with problematic behaviours using simple group comparisons without further reporting analyses adjusted for potential confounding factors that may have contributed to the association.

### PTSD and comorbidity with other mental disorders

PTSD was found to be highly comorbid with other psychiatric disorders (Table 2). Significantly higher rates of psychiatric comorbidity were found amongst those with PTSD compared to those without such a diagnosis although analyses were not always adjusted for potential confounding. Affective disorders, most notably depression (n = 13), and anxiety disorders (n = 9) were the most frequently researched comorbidities in studies of both youth and adult prisoners.

**Table 2:** Associations between PTSD and comorbid mental disorders.

**Study author, year**	**Sampling**	**Country**	**Age range (mean)**	**Male/Female**	**Exposure**	**Measure of Mental Disorder**	**Timing**	**Measure of Association**	**Quality**
**Depression** *Youth*									
Abram, 2007	Random	USA	10–18	531/360	PTSD (past year)	Affective disorders (including depression) measured by diagnostic interview (DISC-IV)	Past year	M: OR = 1.0; 95% CI = 0.3–4.0; NS F: OR = 1.0; 95% CI = 0.5–1.9; NS	38/42 High
Ariga, 2008	Random	Japan	16–19	0/64	PTSD (past month)	Depression measured by diagnostic interview (MINI-Kid)	Past month	Compared to those without PTSD: No significant associations	30/42 Medium
Dixon, 2005	Consecutive	Australia	13–19	0/100	PTSD (Lifetime)	Depression measured by diagnostic interview (K-SADS)	Lifetime	X^2^ value not reported p = .001	29/42 Medium
Ford, 2018	Convenience	USA	12–19 (16.08)	599/210	PTSS (Past month)	“Depressed-anxious” symptoms measured by MAYSI-2	Past month	Mediation analysis showed a direct relationship between PTSD symptoms and depressed-anxious symptoms, B = 0.05, p<0.001	22/42 Medium
Kerig, 2009	Consecutive	USA	10–17	199/90	PTSS (past month); CPTSD symptoms	“Depressed-anxious” symptoms measured by MAYSI-2	Past month	PTSD symptoms: F: r = 0.74, p < .001; M: r = 0.64, p < .001 CPTSD symptoms: F: r = 0.56, p < .001; M: r = 0.54, p < .001	27/42 Medium
Kerig, 2016	Consecutive	USA	13–19	158/63	PTSS (past month)	Depression symptoms measured by Children’s Depression Inventory	Past month	PTSD symptoms: r = 0.43, p < .001	27/42 Medium
Ulzen, 2003	Matched	Canada	13–17	49 ^a^	PTSD (lifetime)	Depression measured by diagnostic interview (DICA-R)	Current; Lifetime	Current depression: M^2^ = 5.75, p < .05 Lifetime depression: M^2^ = 16.55, p < .001	17/42 Low
*Adult*									
Caraballo, 2013	Random	Puerto Rico	18–74	831/181	PTSS (past week)	Depression symptoms measured by CES-D; Diagnosis measured by CIDI	Current (past week; past year)	Symptoms: r = 0.56, p<0.001 Depression diagnosis among those with PTSD: t = 6.10, p < .001	24/42 Medium
Gibson, 1999	Random	USA	(32)	213/0	PTSD (6-month and Lifetime)	Major Depression measured by diagnostic interview (DIS-III).	Current; Lifetime	Current PTSD/Depression: X^2^ = 7.67, p < .01 Lifetime PTSD/Depression: X^2^ = 18.63, p < .01	29/42 Medium
**Study author, year**	**Sampling**	**Country**	**Age range (mean)**	**Male/Female**	**Exposure**	**Measure of Mental Disorder**	**Timing**	**Measure of Association**	**Quality**
Harner, 2015	Voluntary	USA	20–85	0/387	PTSD symptoms (past month)	Depression diagnoses measured by self-report questionnaire (Prison Health Survey)	Lifetime	Those with Moderate to Severe PTSD symptoms, compared to those without symptoms: X^2^ = 25.23, p < .01	19/42 Low
Heffernan, 2015	Random	Australia	(M = 31.49 F = 28.82)	331/65	PTSD (past year)	Depression measured by diagnostic interview (CIDI)	Past year	OR = 5.15, 2.50–10.37, p < .001 *analysis not separated by gender	37/42 High
Perez-Pedrogo, 2018^c^	Random	Puerto Rico	18–74	959/220	PTSS (past week)	Major depression measured by diagnostic interview (UM CIDI)	Lifetime	M: OR = 2.64, 95% CI = 1.07–6.53, p<0.05 F: OR = 3.61, 95% CI = 1.33–9.81, p<0.05	27/42 Medium
Zlotnick, 1997	Random	USA	(31)	0/85	PTSD (Lifetime)	Major depressive episode measured by diagnostic interview (SCID-IV)	Current; Lifetime	Current depression: X^2^ = 12.22, p = .0005 Lifetime depression: Not significant	28/42 Medium
**Anxiety** *Youth*									
Abram, 2007	Random	USA	10–18	531/360	PTSD (Past year)	Anxiety disorders measured by diagnostic interview (DISC-IV)	Past year	M: OR = 3.2, 95%CI = 1.0–10.2; p = .0496 F: OR = 0.8; 95% CI = 0.4–1.6; p = NS	38/42 High
Ariga, 2008	Random	Japan	16–19	0/64	PTSD (past month)	Anxiety disorders measured by diagnostic interview (MINI-Kid)	Past month	Panic disorder: X^2^ = 14.8, p < .001 Separation anxiety: X^2^ = 13.0, p < .01 Social phobia: X^2^ = 17.7, p < .001 Agoraphobia: NS Specific phobia: NS OCD: NS	30/42 Medium
Dixon, 2005	Consecutive	Australia	13–19	0/100	PTSD (Lifetime)	Anxiety disorders measured by diagnostic interview (K-SADS)	Lifetime	Other anxiety disorders: *p* = .02 Panic disorder: *p* = .02 GAD: *p* = .003 Simple/social phobia: NS OCD: NS	29/42 Medium
Ulzen, 2003	Matched	Canada	13–17	49^a^	PTSD (Lifetime)	“Overanxious” disorder measured by diagnostic interview (DICA-R)	Current	M^2^ = 8.25; p < .05; X2 = 4.49, p < .05	17/42 Low
*Adult*									
Caraballo, 2013	Random	Puerto Rico	18–74	831/181	PTSS (Past week)	Generalized anxiety disorder measured by diagnostic interview (CIDI)	Current	*t* = 2.025, *p* = 0.043	24/42 Medium
**Study author and year**	**Sampling**	**Country**	**Age range (mean)**	**Male/Female**	**Exposure**	**Measure of Mental Disorder**	**Timing**	**Measure of Association**	**Quality**
Gibson, 1999	Random	USA	(32)	213/0	PTSD (6 month and Lifetime)	GAD, OCD and Panic Disorder measured by diagnostic interview (DIS-III)	Current Lifetime	Current PTSD/GAD: X^2^ = 6.74, p < .01 Current PTSD/OCD:X^2^ = 6.88, p < .01 Current PTSD/Panic: NS	29/42 Medium
Harner, 2015	Voluntary	USA	20–85	0/387	PTSD symptoms (Past month)	Anxiety diagnoses measured by self-report (Prison Health Survey)	Lifetime	Those with PTSD symptoms (11+) compared to those with none: Anxiety disorder: X^2^ = 38.68, p < .01 Panic attacks: X^2^ = 32.95, p < .01	19/42 Low
Heffernan, 2015	Random	Australia	(M = 31.49 F = 28.82)	331/65	PTSD (Past year)	Other anxiety disorders measured by CIDI	Past year	OR = 3.16, 95% CI 1.57–6.15p < .001 *analysis not separated by gender	37/42 High
Perez-Pedrogo, 2018	Random	Puerto Rico	18–74	959/220	PTSS (Past week)	Generalised anxiety disorder measured by diagnostic interview (UM CIDI)	Lifetime	M: OR = 6.54, 95% CI = 1.50–28.55, p<0.05 F: too few participants to complete analysis	27/42 Medium
**Conduct Disorder, CU Traits** *Youth*									
Ariga, 2008	Random	Japan	16–19	0/64	PTSD (past month)	CD measured by diagnostic interview (MINI-Kid)	Lifetime	No significant differences between those with PTSD and those without	30/42 Medium
Dixon, 2005	Consecutive	Australia	13–19	0/100	PTSD (Lifetime)	CD measured by diagnostic interview (K-SADS)	Lifetime	No significant differences between those with PTSD and those without	29/42 Medium
Kimonis, 2011	Convenience	USA	(16.43)	182^b^/0	PTSS (Lifetime)	CU traits measured by validated self-report questionnaire (ICU)	Current	No correlation between PTSD symptoms and CU traits	30/42 Medium
Sharf, 2014	Convenience	USA	14–19	238/0	PTSS (past month)	CU traits measured by validated self-report questionnaire (ICU) Participants then categorized into three groups: non-psychopaths (n = 149) primary (n = 43), and secondary (n = 44) CU variants.	Current	Total scores: r = 0.15, p < .05 Youth with PTSD scored higher on callousness subscale scores compared to those not meeting criteria: t(24.89) = -2.33, p = 0.028. Means did not differ on ICU total score or other subscales (uncaring, unemotional). *Differences in PTSD symptom scores between groups*: Total PTSD symptoms (F = 8.61, p < .001). Post hoc comparisons: Secondary CU group > Primary CU group (p = .004). Secondary CU group > Nonpsychopathic group (p < .001).	29/42 Medium
**Study author and year**	**Sampling**	**Country**	**Age range (mean)**	**Male/Female**	**Exposure**	**Measure of Mental Disorder**	**Timing**	**Measure of Association**	**Quality**
Ulzen, 2003	Matched	Canada	13–17	49^a^	PTSD (Lifetime)	CD and Oppositional Defiance Disorder (ODD) measured by diagnostic interview (DICA-R)	Lifetime	No significant differences between those with PTSD and those without.	17/42 Low
**PD, Psychopathy** *Adult*									
Gobin, 2015	Convenience	USA	(38.92)	37/51	PTSS (past month)	Antisocial PD measured by diagnostic interview (SCID-IV). Psychopathy symptoms measured by self-report (PPI)	Lifetime	ASPD: AOR = 1.00, p = NS Psychopathy: B = 0.15, p = NS	20/42 Low
Harner, 2015	Voluntary	USA	20–85	0/387	PTSS (past month). Cut-off 11+	Borderline PD diagnosis measured by self-report questionnaire (Prison Health Survey)	Lifetime	X^2^ = 23.93, p < .01	19/42 Low
Warren, 2009	Convenience	USA	(33.2)	0/201	PTSD (Current)	PD symptoms measured using a diagnostic interview (SCID-IV)	Lifetime	Schizoid PD symptoms: t = 2.47, p < .05 Borderline PD symptoms: t = 2.32, p < .05 Avoidant PD symptoms: t = 2.08, p < .05	26/42 Medium
Willemsen, 2012	Voluntary	Belgium	20–73	81/0	PTSS (Lifetime)	Psychopathy measured by semi-structured diagnostic interview (PCL-R)	Lifetime	Associations between PTSD symptoms and PCL-R Total Score: *B* = -.026, X^2^ = 2.74, p < .05	26/42 Medium
Woodfield, 2017	Voluntary	UK	18–61	101/0	PTSS (Past month)	Psychopathy measured by self-report questionnaire (Self-Report Psychopathy Scale, Short Form)	Lifetime	Primary psychopathy: r = 0.23, p < .05 Secondary psychopathy: r = 0.32, p < .01	20/42 Low
Zlotnick, 1997	Random	USA	(31)	0/85	PTSD (Lifetime)	ASPD and BPD measured by diagnostic interview (SCID-IV)	Lifetime	BPD: X^2^ = 8.15, p = .004 ASPD: NS	28/42 Medium
**Psychosis** *Youth*									
Ariga, 2008	Random	Japan	16–19	0/64	PTSD (past month)	Psychotic disorder measured by diagnostic interview (MINI-Kid)	Past month; Lifetime	Current psychotic disorder: NS Lifetime psychotic disorder: X^2^ = 8.0, p < .05	30/42 Medium
Dixon, 2005	Consecutive	Australia	13–19	0/100	PTSD (Lifetime)	Psychotic disorder diagnoses measured by diagnostic interview (K-SADS)	Lifetime	X^2^ not reported, p < .008	29/42 Medium
*Adult*									
**Study author and year**	**Sampling**	**Country**	**Age range (mean)**	**Male/Female**	**Exposure**	**Measure of Mental Disorder**	**Timing**	**Measure of Association**	**Quality**
Gibson, 1999	Random	USA	(32)	213/0	PTSD (6 month and lifetime)	Schizophrenia and Schizoaffective disorder measured by diagnostic interview (DIS-III)	Current; lifetime	No significant differences between those with or without PTSD in rates of psychotic disorder	29/42 Medium
Harner, 2015	Voluntary	USA	20–85	0/387	PTSS (past month). Cut-off 11+	Schizophrenia diagnosis measured by self-report, using the Prison Health Survey	Lifetime	No significant associations	19/42 Low
Heffernan, 2015	Random	Australia	(M = 31.49 F = 28.82)	331/65	PTSD (past year)	Psychotic disorder diagnosis measured by diagnostic interview (CIDI)	Past year	OR = 4.04, CI 1.83–8.63, p < .001 *not reported separately by gender	37/42 High
**ADHD** *Youth*									
Abram, 2007	Random	USA	10–18	531/360	PTSD (Past year)	ADHD or other behavioural disorder (including CD and ODD) measured by diagnostic interview (DISC-IV). Disorders combined for analysis	Past year	M: OR = 0.9, CI 0.3–2.8, p = 0.85 F: OR = 0.9, CI 0.3–2.8, p = 0.85	38/42 High
Ariga, 2008	Random	Japan	16–19	0/64	PTSD (Past month)	ADHD measured by diagnostic interview (MINI-Kid)	Past month	No significant differences	30/42 Medium
Dixon, 2005	Consecutive	Australia	13–19	0/100	PTSD (Lifetime)	ADHD and CD measured by diagnostic interview (K-SADS)	Lifetime	No significant differences	29/42 Medium
Ulzen, 2003	Matched	Canada	13–17	49^a^	PTSD (Lifetime)	ADHD measured by diagnostic interview	Lifetime	Comorbid ADHD: M^2^ = 0.79, p = NS	17/42 Low
Perez-Pedrogo, 2018	Random	Puerto Rico	18–74	959/220	PTSS (Past week)	Antecedents of childhood ADHD measured using the Spanish version of the Wender Utah Rating Scale	Childhood	M: OR = 4.71, 95% CI = 2.50–8.88, p<0.001 F: OR = 3.36, 95% CI = 1.50–7.51, p<0.05	27/42 Medium
*Adult*									
Moore, 2016	Random	Australia	(31)	67/21	PTSD (Past month)	Screened for ADHD symptoms using a validated self-report questionnaire (ASRS). Adult ADHD then measured by diagnostic interview (MINI Plus).	Past six months	OR = 3.89, CI 1.01 14.95, p < .05 AOR = 2.76, CI 0.63–12.02, p = NS	25/42 Medium
**Substance, Alcohol Use** *Youth*									
Abram, 2007	Random	USA	10–18	531/360	PTSD (past year)	Drug-Use and Alcohol Use Disorders measured by diagnostic interview (DISC-IV)	Past year	Comorbid DUD: Males: OR = 3.6, CI = 1.2–11.1, p = .03 Females: OR = 1.7, CI = 0.9–3.2, p = .07 Comorbid AUD: Males: OR = 2.9, CI 1.0–8.6, p = .049 Females: OR = 2.2, CI 1.2–4.2, p = .01	38/42 High
**Study author and year**	**Sampling**	**Country**	**Age range (Mean age)**	**Male/Female**	**Exposure**	**Measure of Outcome**	**Timing**	**Measure of Association**	**Quality**
Ariga, 2008	Random	Japan	16–19	0/64	PTSD (past month)	Substance abuse, dependence and alcohol abuse, dependence measured by diagnostic interview (MINI-Kid)	Past month	No significant differences in rates between those with PTSD and those without	30/42 Medium
Dixon, 2005	Convenience	Australia	13–19	0/100	PTSD (Lifetime)	“Substance and alcohol abuse/dependence” measured by diagnostic interview (K-SADS)	Lifetime	Those with PTSD, compared to those without: X^2^ value not provided; p = .02	29/42 Medium
Ford, 2018	Convenience	USA	12–19 (16.08)	599/210	PTSS (past month)	“Alcohol/drug use” measured by self-report questionnaire (MAYSI-2)	Current	No evidence of a direct association between PTSD and alcohol/drug use in mediation analysis	22/42 Medium
Kerig, 2009	Convenience	USA	10–17	253/38	PTSS (past month) CPTSD symptoms	“Alcohol/drug use” measured by self-report questionnaire (MAYSI-2)	Current	PTSD symptoms: r = 0.34, p < .001 CPTSD symptoms: r = 0.23, p < .001	27/42 Medium
Moore, 2013	Convenience	Australia	13–21	314/47	PTSD (Lifetime)	Use of illicit drugs (weekly) prior to incarceration; measured by self-report	Current	OR = 2.78, CI 1.45–5.32, p < .05 AOR = 2.16, CI 0.99–4.72, p = NS	28/42 Medium
Ulzen, 2003	Matched	Canada	13–17	49 (gender distribution not reported)	PTSD (Lifetime)	Substance and alcohol use disorders measured by diagnostic interview (DICA-R)	Current	Substance use disorders: No significant differences between those with PTSD and those without Alcohol use disorders: M^2^ = 4.45, p < .05	17/42 Low
*Adult*									
Giarrantano, 2017	Random	USA	(31.64)	301/196	Complex PTSD (Lifetime)	Risk of problematic substance use measured by screening tool (ASSIST). Dichotomized into Low and Moderate/High risk	Current	Relationship between CPTSD symptoms and Drug Risk, when adjusting for gender: OR = 1.03, CI 1.01–1.05, p < .001	37/42 High
Gibson, 1999	Random	USA	(32)	213/0	PTSD (6 months; Lifetime)	Drug, Alcohol abuse/dependence	Lifetime	No significant differences between those with or without PTSD in rates of Drug or Alcohol abuse/dependence	29/42 Medium
**Study author and year**	**Sampling**	**Country**	**Age range (Mean age)**	**Male/Female**	**Exposure**	**Measure of Outcome**	**Timing**	**Measure of Association**	**Quality**
Harner, 2015	Voluntary	USA	20–85	0/387	PTSS (past month) symptom cut-off 11+)	Substance, Alcohol addiction diagnoses measured by self-report (Prison Health Survey)	Lifetime	No significant differences between those with Moderate or above symptoms on any alcohol or substance use outcome	19/42 Low
Heffernan, 2015	Random	Australia	(M = 31.49 F = 28.82)	331/65	PTSD (Past year)	Substance and Alcohol Use Disorders measured by diagnostic interview	Past year	Cannabis: OR = 2.14, CI 1.12–4, p < .05 Opiates, Sedatives, Amphetamines: NS Alcohol: NS	37/42 High
Howard, 2017b	Convenience	UK	(34.52)	0/89	PTSS (Past month)	History of drug use assessed by single dichotomous self-report question	Lifetime	*B* = .05, OR = 1.05, CI = 1.02–1.08, *p value not provided*	25/42 Medium
Perez-Pedrogo, 2018	Random	Puerto Rico	18–74	959/220	PTSS (Past week)	Substance misuse disorder measured by diagnostic interview (UM CIDI)	Lifetime	M: OR = 1.72 95% CI = 1.05–2.84, p<0.05 F: OR = 1.26, 95% CI = 0.31–5.12, NS	27/42 Medium
**Any Other Psychiatric Disorder** *Youth*									
Abram, 2007	Random	USA	10–18	531/360	PTSD (past year)	Comorbid psychiatric disorders measured by diagnostic interview (DISC-IV)	Past year	OR(95% CI) = 7.3 (3.2–16.5); p<. 001 M: OR(95%) = 9.0 (3.4–23.7); p < .001 F: OR(95%) = 1.6 (0.7–3.5); p = 0.22	38/42 High
Dixon, 2005	Consecutive	Australia	13–19	0/100	PTSD (Lifetime)	Psychiatric diagnosis measured by diagnostic interview (K-SADS)	Lifetime	4 or more disorders: OR(95% CI) = 19.71 (5.44–71.43); p < .001 AOR(95%) = 14.48 (3.73–56.27); p < .001	29/42 Medium
Moore, 2013	Convenience	Australia	13–21	253/38	PTSD (“present” and lifetime)	Psychiatric diagnosis measured by diagnostic interview (K-SADS)	Lifetime	2 or more disorders OR(95% CI) = 4.90 (2.59–9.28); p < .001. AOR(95% CI) = 3.52 (1.55–7.99); p < .05	28/42 Medium
*Adult*									
Giarrantano, 2017	Random	USA	(M = 30.62, F = 32.30)	301/196	C-PTSD symptoms (Lifetime)	Number of psychiatric diagnoses (excluding PTSD) measured by diagnostic interview (SCID-IV)	Current	*B* = 0.10, 95% 0.09–0.11, p < .001	37/42 High
Heffernan, 2015	Random	Australia	(M = 31.49 F = 28.82)	331/65	PTSD (past year)	Psychiatric disorders measured by diagnostic interview (CDI)	Past year	OR = 2.42, 95% CI [1.12, 5.80], p = .022.	37/42 High

^a^ Gender distribution of sample not reported

^b^ CU traits only measured within a subsample of total study sample (N = 373)

^c^ Includes sample from Caraballo, 2013

#### Depression

**Fig 2:**
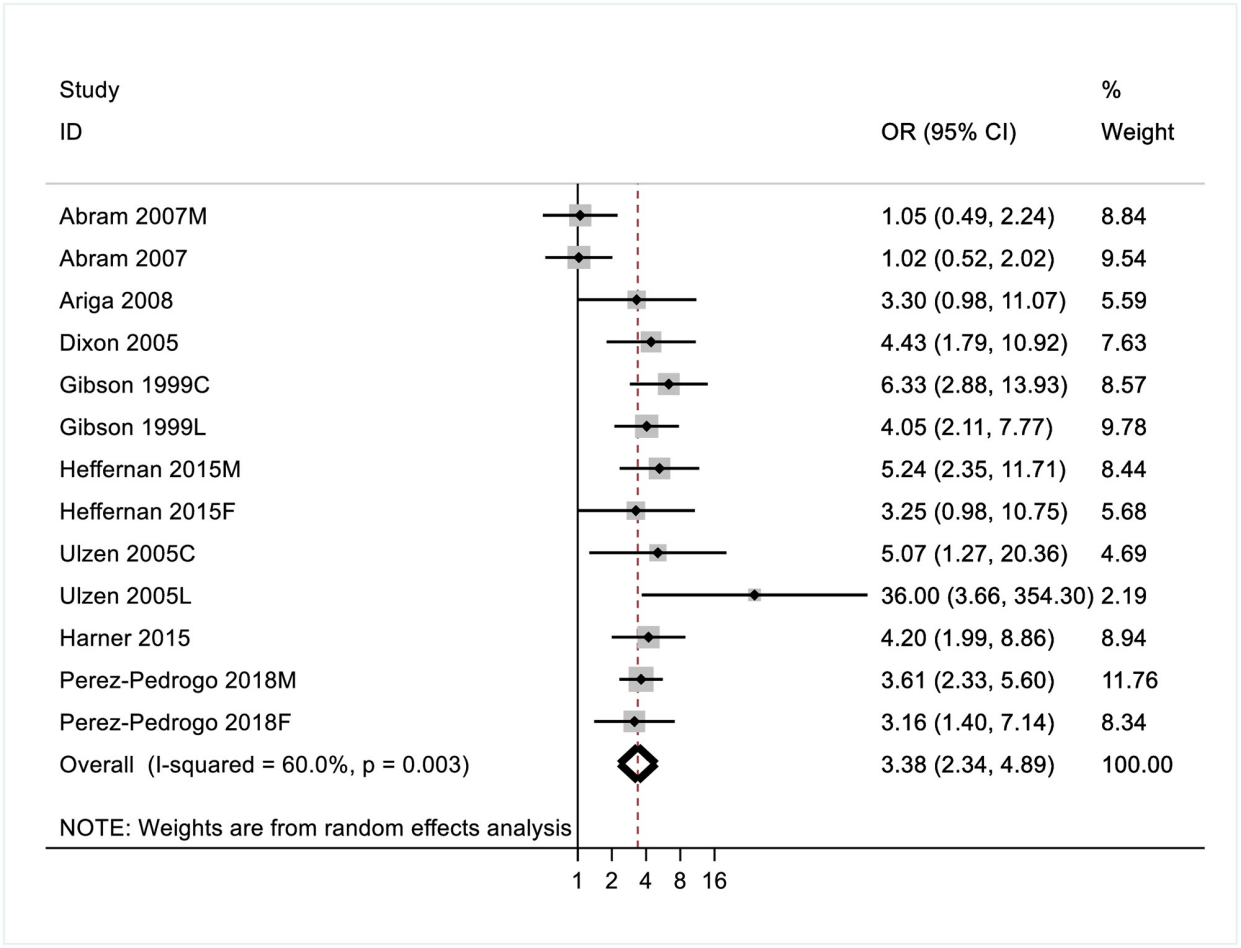
Odds ratios (ORs) for the association between PTSD and comorbid depressive disorders.

Across the eight studies included in the depression domain, the random-effects pooled OR of a comorbid depressive disorder was 3.4 (95% CI [2.34, 4.89]) in individuals with PTSD (Fig 2).

There was substantial heterogeneity between studies (I^2^ = 60.0%). We therefore analysed results by subgroup, to explore how estimates were affected by gender, age and timing of PTSD diagnosis.

Gender. Differences in risk estimates of comorbidity with depression were observed for females (pooled OR 2.81, 95% CI [1.65–4.78]) compared to males (pooled OR 3.48, 95% CI [2.03–5.96]). Significant heterogeneity was observed for the male (I^2^ = 69.3%), but not the female (I^2^ = 53.2%, p = 0.058) subgroups.

Age. Differences in risk estimates were also found for incarcerated youth (pooled OR 2.70 CI [1.24–6.26]) and adult prisoners (pooled OR = 4.07, CI [3.13–5.28]). Significant heterogeneity was observed for the youth subgroup (I^2^ = 72.2%), but not the adult group.

Timing of PTSD Diagnosis. Risk estimates of comorbidity with depression were higher for lifetime PTSD (pooled OR = 5.17 CI[2.42–10.99]) than current PTSD (pooled OR = 2.96 CI[1.66–5.25]). Significant heterogeneity was observed within the current PTSD subgroup (I^2^ = 63.1%) but not for lifetime PTSD.

#### Generalized anxiety disorder

**Fig 3:**
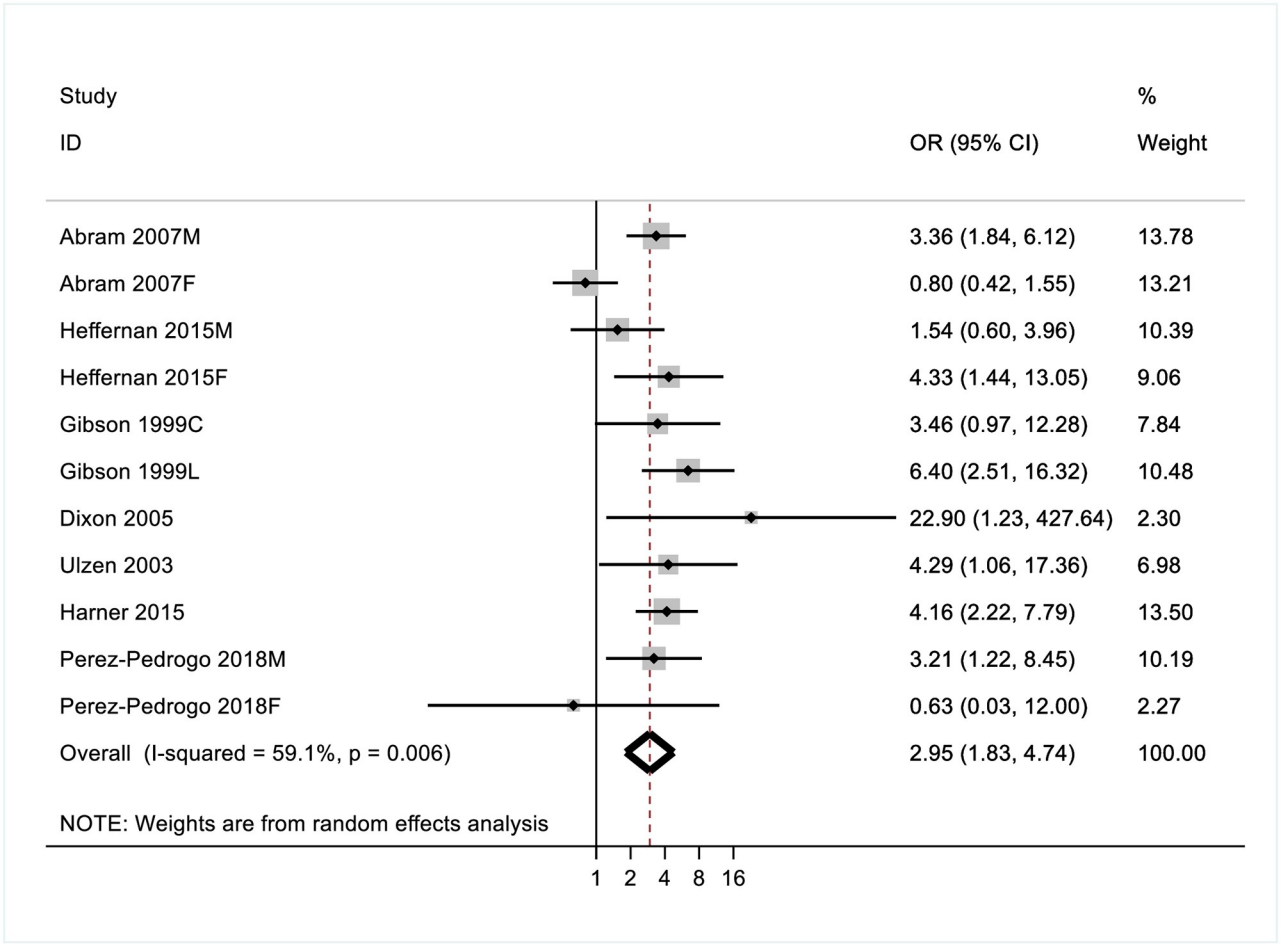
Odds ratios (ORs) for the association between PTSD and comorbid anxiety disorder(s).

Across the eight studies included in the GAD domain, the random-effects pooled OR of a comorbid anxiety disorder was 2.43 (95% CI [1.19,4.96]) in individuals with PTSD. Substantial heterogeneity was observed (I^2^ = 79.8%). After removing one study from the meta-analysis due to an effect size that was an outlier to the group [27], the observed Odds Ratio for comorbidity between PTSD and Anxiety was 2.95 (95% CI [1.83–4.74]) (Fig 3). Heterogeneity estimates reduced but remained significant (I^2^ = 59.1%). We therefore analysed results by subgroup, to explore how estimates were affected by gender, age and timing of PTSD diagnosis.

Gender. Risk estimates of comorbidity with anxiety were higher for male (OR = 3.27, CI [1.91–5.62]) than female (OR = 2.58, CI [0.95–9.58]) prisoners. Significant heterogeneity was observed within the female (I^2^ = 77.3%), but not the male subgroup (I^2^ = 9.9%, p = 0.35).

Age. Differences in risk estimates were observed for adult prisoners (OR = 3.52, CI [2.41–5.15]) and incarcerated youth (OR = 2.70, CI [0.90–8.16]). Significant heterogeneity was observed for the youth (I^2^ = 78.8%), but not the adult subgroup (I^2^ = 4.3%, p = 0.39).

Timing of PTSD. Differences in risk estimates for GAD comorbidity were also observed for current (OR = 2.43, CI [1.45–4.09]) and lifetime (OR = 6.20, CI [2.92–13.15]) PTSD. Significant heterogeneity was observed for the current PTSD subgroup (I^2^ = 61.7%) but not the lifetime diagnostic subgroup.

#### Substance use disorder

**Fig 4:**
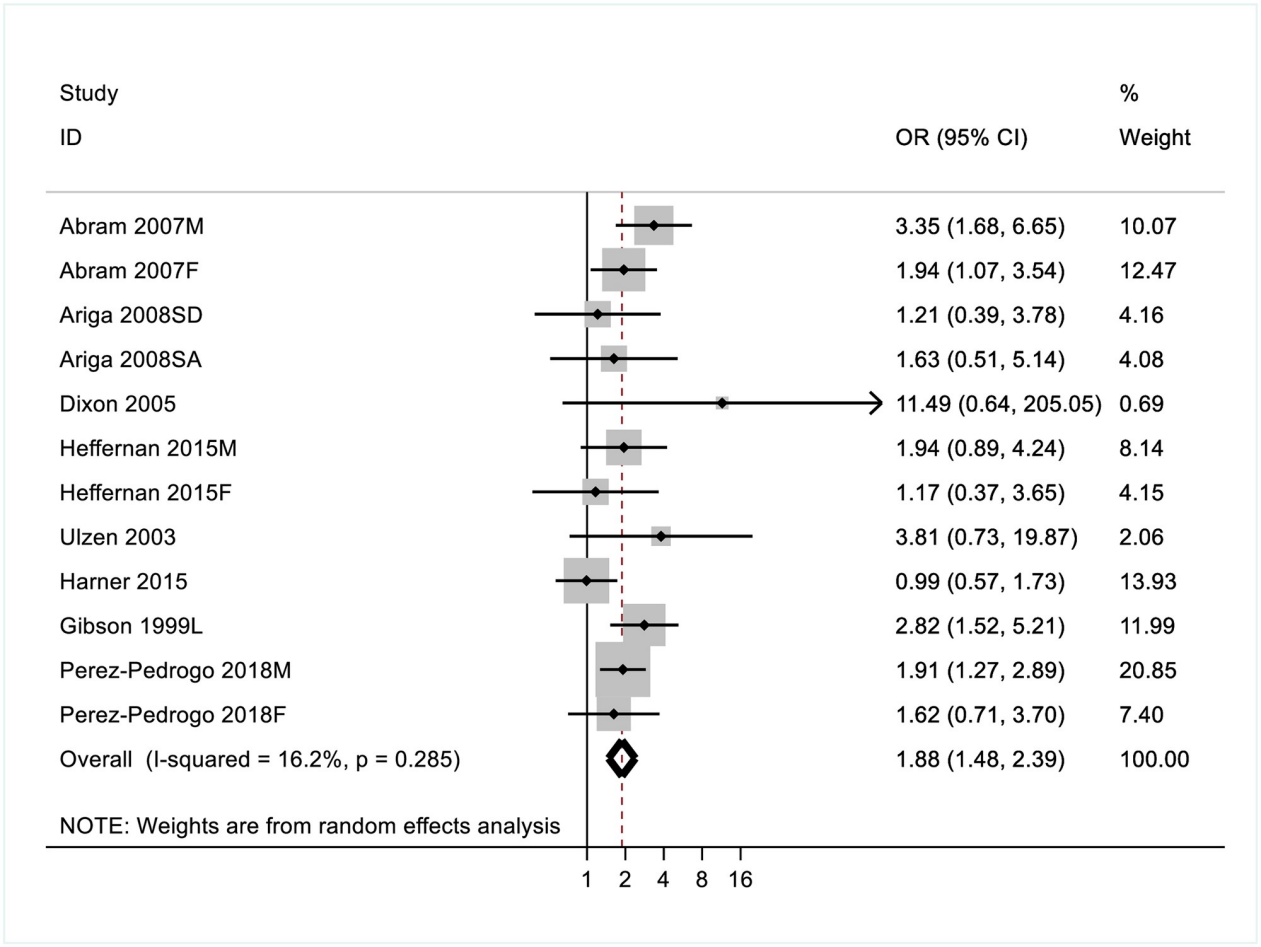
Odds ratios (ORs) for the association between PTSD and comorbid substance use disorder(s).

Fifteen studies examined the association between PTSD and substance misuse, three of which were high quality. Inconsistent evidence of an association between PTSD and alcohol misuse among both youth (n = 4) and adult (n = 5) samples (Table 2). Across the seven studies included in the substance use domain, the random-effects pooled OR of a comorbid substance use disorder was 1.91 (95%CI [1.38–2.66]) in individuals with PTSD (Fig 4). No significant heterogeneity was observed for this disorder (I^2^ = 30.8%, p = 0.163). Differences in risk estimates of SUD were observed for males (OR = 2.31, CI [1.73–3.05) and females (OR = 1.42, CI [1.03–1.95). Differences in risk estimates for comorbid SUD were also observed for incarcerated youth (OR = 2.28, CI [1.56–3.34]) and adult prisoners (OR = 1.70, CI [1.24–2.33]); and for current (OR = 1.72, CI [1.35–2.20]) and lifetime PTSD (OR = 3.08, CI [1.75–5.42]).

#### Psychosis

Five studies examined psychotic illnesses, and three found statistically significant associations. However, studies reporting positive associations included two small samples of female youth [27, 30] and one highly selected sample of adult Aboriginal prisoners [46]. Of the five studies examining associations between PTSD and ADHD, one found a statistically significant association [59].

#### Personality disorder

PTSD and Personality Disorder (PD), were found to be comorbid in four of five adult studies. Among samples of incarcerated females (n = 3), PTSD was found to be most consistently and strongly associated with Borderline PD. One medium quality study of male prisoners found lifetime PTSD to be associated with ASPD [43]. No study found statistically significantly elevated rates of PTSD among prisoners with primary psychopathy compared to those without; one medium-quality study found that these two disorders were negatively associated. Studies of the developmental precursors of these disorders (conduct disorder, callous unemotional traits) suggested mixed associations with PTSD. While one medium-quality study [34] found no association between PTSD and CU trait scores, another similar quality study, also of detained male youth [36], found that those with PTSD had significantly higher callousness trait scores than those without.

### PTSD and its relationship with problematic behaviours

#### Suicidality

**Fig 5:**
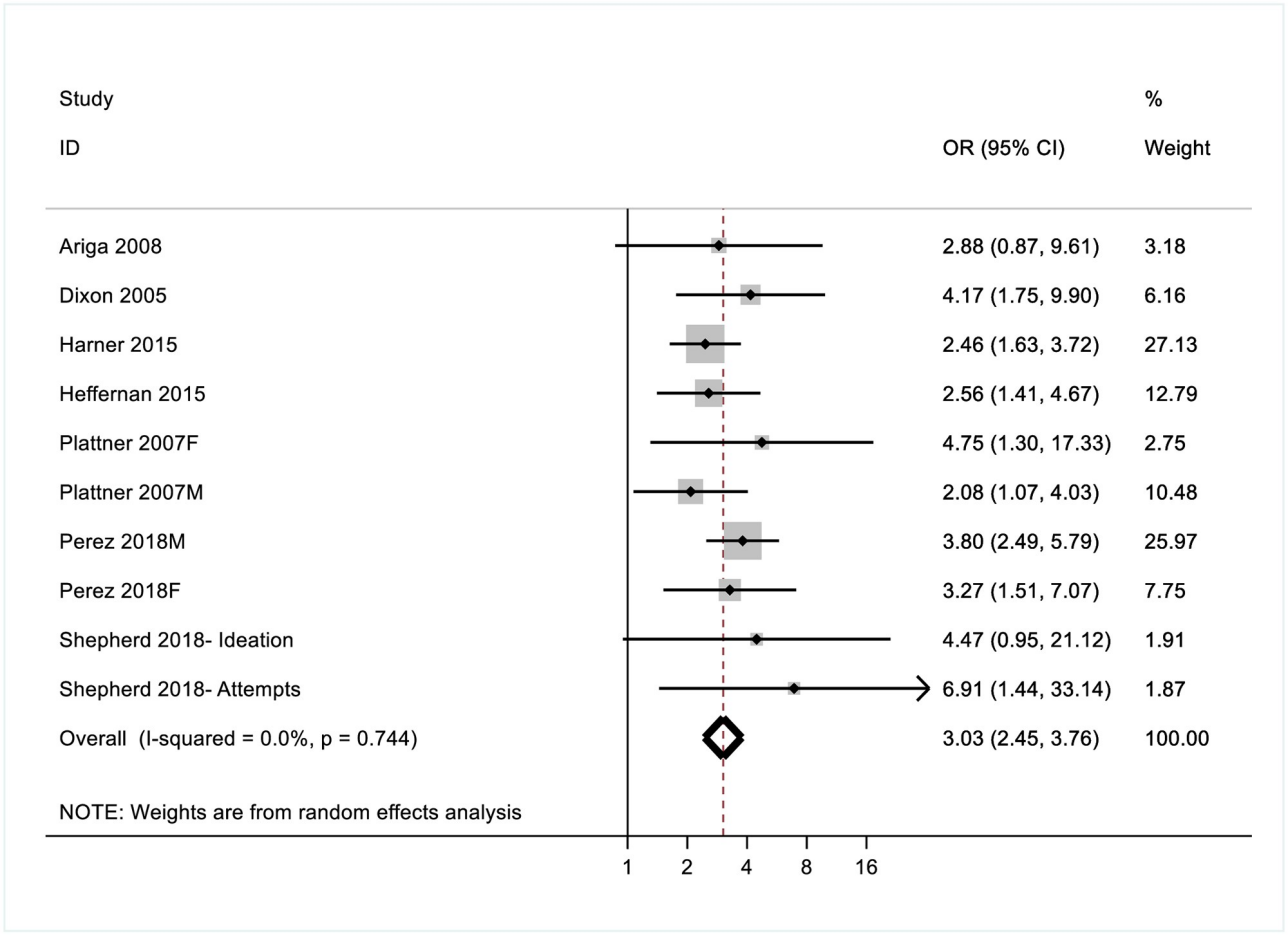
Odds ratios (ORs) for the association between PTSD and suicidality.

Across the seven studies included in the suicidality domain (which included lifetime suicide attempts and current risk of suicidal behaviour), the random-effects pooled OR was 3.03 (CI 2.45–3.76)–see Fig 5. No significant heterogeneity was detected in the overall model. Minimal differences in risk estimates were observed for youth (OR = 2.91, CI[1.85–4.57]) and adult (OR = 3.07, CI[2.40–3.92) participants; some differences in risk estimates were observed for male (OR = 3.34, CI[2.28–4.88]) and female (OR = 2.90, CI[2.12–3.97]) participants.

In total, twelve studies investigated the association between PTSD and problems relating to suicidality, which included suicide attempts, suicidal ideation, measures of suicide risk, or self-injurious behaviour, and nine found statistically significant associations (Table 3). Three studies investigated associations between PTSD and non-suicidal self-injury (NSSI), with all three also reporting positive main effects. However, most studies examining suicidality made simple group comparisons (e.g. PTSD vs no PTSD) and did not account statistically for other covariates which may have accounted for all or part of the observed association. Four studies (29, 37, 58, 59) conducted multivariate analyses, and found that positive univariate associations between PTSD and NSSI or suicidality were rendered non-significant once added to a multivariate model with other significant correlates of PTSD such as childhood maltreatment or psychiatric comorbidity.

**Table 3:** Associations between PTSD and problematic behaviours.

**Study author, year**	**Sampling**	**Country**	**Study Design**	**Age range (Mean age)**	**Male/Female**	**Exposure**	**Measure of Outcome**	**Timing**	**Measure of Association**	**Quality**
**Suicidality** *Youth*										
Ariga, 2008	Random	Japan	Cross sectional	16–19	0/64	PTSD (Past month)	Risk of suicide measured by diagnostic interview (MINI-Kid)	Past month	No significant differences between those with and without PTSD	30/42 Medium
Dixon, 2005	Convenience	Australia	Cross sectional	13–19	0/100	PTSD (Lifetime)	History of suicide attempts measured by diagnostic interview (K-SADS)	Lifetime	OR: 4.17, CI 1.8–9.9 AOR: NS	29/42 Medium
Ford, 2018	Convenience	USA	Cross sectional	12–19 (16.08)	599/210	PTSS (Past month)	Suicidal ideation measured by a subscale of a screening questionnaire (MAYSI-2)	Current	Mediation analysis showed a direct relationship between PTSD symptoms and suicidal ideation, B = 0.02, p<0.001.	22/42 Medium
Kerig, 2009	Consecutive	USA	Cross sectional	10–17	199/90	PTSS (current)	Suicidal ideation measured by a subscale of a screening questionnaire (MAYSI-2)	Current	PTSD symptoms: Males: r = 0.38, p < .001 Females: r = 0.57, p < .001 CPTSD symptoms: Males: r = 0.29, p < .001 Females: r = 0.57, p < .001	24/42 Medium
Moore, 2013	Convenience	Australia	Cohort	13–21	253/38	PTSD (Lifetime)	History of self-harm and suicide attempts measured	Lifetime	Self-harm: OR = 2.85, CI 1.4–6.0 AOR = 1.19, CI 0.4–3.6 Suicide attempts: OR = 3.0, CI 1.3–6.9 AOR = 0.79, CI 0.2–2.6	28/42 Medium
Plattner, 2007	Convenience	Austria	Cross sectional	14–21	266/53	PTSD (past month)	Suicidality risk measured by diagnostic interview (MINI-Kid)	Past month	Males: X^2^ = 4.8, p = .028 Females: X^2^ = 6.0, p = .014	27/42 Medium
*Adult*										
Caraballo, 2013	Random	Puerto Rico	Cross sectional	18–74	831/181	PTSS (past week)	History of suicide attempts measured by dichotomous self-report item	Lifetime	Prevalence of lifetime suicide attempts among those with PTSD, compared to those without: t = 4.467, p < .001	24/52 Medium
Harner, 2015	Voluntary	USA	Cross sectional	20–85	0/387	PTSS (symptom cut-off 11+)	History of suicide attempts and self-injury measured by self-report questionnaire (Prison Health Survey)	Lifetime	History of lifetime suicide attempts: X^2^ = 25.97, p < .01 History of suicide attempts in prison: Not significant History of self-injury in prison: LR = 11.12, p < .05	19/42 Medium
**Study author, year**	**Sampling**	**Country**	**Study Design**	**Age range (mean)**	**Male/Female**	**Exposure**	**Measure of Outcome**	**Timing**	**Measure of Association**	**Quality**
Heffernan, 2015	Random	Australia	Cross sectional	(M = 31.49, F = 28.82)	331/65	PTSD (past year)	Suicidal ideation and history of suicide attempts measured by diagnostic interview (CIDI)	Past 12 months; Lifetime	Suicidal ideation (Lifetime): OR = 2.43, CI 1.3–4.4 Suicidal ideation (12 month): OR = 3.58, CI 1.6–7.5 Suicidal ideation (current): OR = 3.41, CI 0.5–18.0 Suicide attempts: OR = 2.56, CI 1.3–4.8	37/42 High
Howard, 2017a	Convenience	UK	Cross sectional	(34.52)	0/89	PTSS (past month)	History of self-harm measured by dichotomous self-report item	Lifetime	t = -2.58, p < .05	25/42 Medium
Perez-Pedrogo, 2018	Random	Puerto Rico	Cross sectional	18–74	959/220	PTSS (Past week)	Suicidal thoughts and attempts measured using validated checklist (CES-D)	Past week	M: aOR = 1.84, 95% CI = 0.9–3.9 F: aOR = 1.06, 95% CI = 0.3–3.4	27/42 Medium
Shepherd, 2018	Convenience	Australia	Cross sectional	18–62 (34.2)	107/0	PTSD (Past month)	Suicidal ideation and suicide attempts measured using dichotomous self-report	Lifetime; Past 12 months	Lifetime suicidal ideation: OR = 4.5 95% CI = 1.1–29.8 Suicidal ideation (12 months): OR = 2.4 95%CI = 0.7–8.1 Suicide attempts (Lifetime): OR = 6.9 95% CI = 1.7–46.5	27/42 Medium
**Violence, Aggression** *Youth*										
Hamerlynck, 2008	Convenience	Netherland	Cross sectional	12–18	0/216	PTSS (past month). Cut-off 40+	Aggressive acts assessed using items from a diagnostic interview (K-SADS)	Past year	Those in “Severe” aggression subgroup, compared to Non- and Mild aggression groups, showed higher levels of PTSS: X^2^ = 12.36; p = .002	22/42 Medium
Kimonis, 2011	Convenience	USA	Cross sectional	(16.43)	373/0	PTSS (Lifetime)	Aggression, violent offending, and institutional violence measured by self-report questionnaires (SAI^a^ and SOS^b^). Infractions measured by file search.	Lifetime; Current sentence	No significant associations for any violent/aggressive outcome	30/42 High
Moore, 2013	Convenience	Australia	Cohort	13–21	253/38	PTSD (Lifetime)	Violent Index offence measured by search of juvenile justice records	Current	OR = 1.23 95%CI = 0.57–2.65	28/42 Medium
**Study author, year**	**Sampling**	**Country**	**Study Design**	**Age range (mean)**	**Male/Female**	**Exposure**	**Measure of Outcome**	**Timing**	**Measure of Association**	**Quality**
Stimmel, 2014	Convenience	USA	Cross sectional	12–16	66/0	PTSS (past month)	Aggression measured by self-report questionnaire (Peer Conflict Scale). Categorized into reactive and proactive aggression	Current	Association between PTSD symptom severity with reactive aggression, after adjusting for traumatic exposure type (community violence): B = 0.27, p = .03 No significant associations between PTSD symptoms and proactive aggression	28/42 Medium
*Adult*										
Collins, 1990	Consecutive	USA	Cross sectional	Adult sample (age distribution not reported)	1140/0	PTSD (Lifetime)	Violent offending measured by Index Offence and arrest history, via prison records	Lifetime	Those diagnosed with PTSD, compared to those without: Incarcerated for homicide, rape or assault (OR = 4.58, p < .001) Arrest History for homicide, rape or assault (OR = 2.05, p < .10) Arrested for violent offence in past year (OR = 6.75, p < .001)	27/42 Medium
Howard, 2017b	Convenience	UK	Cross sectional	18–65	0/89	PTSS (past month). Cut-off 33+	Violent offending measured by dichotomous self-report question: “Have you ever been charged/convicted of a violent offence”	Lifetime	*B* = 0.04, OR = 1.04, CI 1.02, 1.06	25/42 Medium
Wahlstrom, 2015	Convenience	USA	Cross sectional	(34.4)	60/0	PTSS (past month)	Physical aggression measured by modified self-report questionnaire (Conflict Tactic Scale)	Past 3 months	Risk of Aggression Perpetration among those with PTSD: B = 0.37, p = 0.001	19/42 Low
McCallum, 2018	Random	UK	Cross sectional	18–40	126/0	PTSS (Current)	Custodial violent incidents and violent conviction measured by self-report	Lifetime	Custodial violence: B = -1.027, 95% CI = 0.1–0.9, p = 0.043 Violent convictions X^2^ = 0.345, p = 0.557.	20/42 Low
Warren, 2009	Convenience	USA	Cross sectional	(33.2)	0/201	PTSD (Current)	Violent index offence measured by prison records. Prison infractions (violent/non-violent) measured by both prison file review and self-report (Prison Violence Inventory)	Unclear	No significant differences between those with and without PTSD on any measure of violent or aggressive behaviour (No stats given).	26/42 Medium
**Study author, year**	**Sampling**	**Country**	**Study Design**	**Age range (mean)**	**Male/Female**	**Exposure**	**Measure of Outcome**	**Timing**	**Measure of Association**	**Quality**
**Anger** *Youth*										
Ford, 2018	Convenience	USA	Cross sectional	12–19 (16.08)	599/210	PTSS (past month)	Anger-irritability measured by a subscale of a screening questionnaire (MAYSI-2)	Current	Mediation analysis showed a direct relationship between PTSD symptoms and anger-irritability, B = 0.05, p<0.001	22/42 Medium
Huang, 2006	Random	China	Cross sectional	16–54	0/471	PTSD (Past month; Lifetime)	“Anger/hostility” measured by validated self-report questionnaire (SCL-90-R).	Past week	Higher levels of PTSD symptoms associated with higher levels of anger/hostility: F_2,471_ = 27.38, p < .001	37/42 High
Kimonis, 2011	Convenience	USA	Cross sectional	14–17	373/0	PTSS (Lifetime)	Anger measured by validated self-report questionnaire (Novaco Anger Scale).	Past 6 months	Correlations between PTSD symptoms and anger: r = 0.12, p < .05	30/42 Medium
Kerig, 2009	Consecutive	USA	Cross sectional	10–17	199/90	PTSS (past month)	“Anger-Irritability” measured by validated self-report questionnaire (MAYSI-2)	Current	Total PTSD symptom severity: r = 0.54, p < .001 Complex PTSD symptoms: r = 0.42, p < .001	27/42 Medium
*Adult*										
Warren, 2009	Convenience	USA	Cross sectional	(33.2)	0/201	PTSD (current)	Anger measured by the Spielberger Trait Anger subscale	Current	t = 2.27, p < .05	26/42 Medium
**Offending behaviour** *Youth*										
Becker, 2011	Convenience	USA	Cross sectional	12–17	83/0	PTSS (Past month)	Frequency of arrests and severity of charges measured through official records. Charges categorised by rank sum (score range 1–6) to provide a total delinquency severity score.	Past year and lifetime	Number of arrests (Lifetime): B = 0.27, t = 2.33, p < .05 Number of arrests (Past year): B = 0.29, t = 2.63, p < .05 Delinquency severity (Lifetime): B = 0.20, t = 1.64, p = 0.105 Delinquency severity (Past year): B = 0.29, t = 2.63, p < .05	27/42 Medium
Moore 2013	Convenience	Australia	Cross sectional	Dichotomous measure of over or under 16	253/38	PTSD (Lifetime)	Three or more previous incarcerations, and re-incarceration both assessed using the Juvenile Justice database	Lifetime; 18 months post interview	Previous incarcerations: OR = 1.62, CI 0.88–2.98, p = NS Re-incarceration: OR = 2.01, CI 1.10–3.7 aOR = 2.00, CI 0.9–4.2	28/42 Medium
**Study author, year**	**Sampling**	**Country**	**Study Design**	**Age range (mean)**	**Male/Female**	**Exposure**	**Measure of Outcome**	**Timing**	**Measure of Association**	**Quality**
*Adult*										
Ardino, 2013	Convenience	2013	Cross sectional	(M = 33.98, F = 44.36)	50/25	PTSS (Past month)	Re-offending risk measured by standardized self-report risk assessment instrument (IORNS). Consists of three subscales: Static, Dynamic and Protective factors	Current	Static Risk Index: r = 0.22, p = NS Dynamic Risk Index: r = 0.40, p < .01 Protective Strengths Index: r = -0.16, p = NS Total Risk Index: r = 0.33, p = NS	20/42 Low
Karatzias, 2017	Convenience	2017	Cross sectional	(41)	0/89	PTSS (Past month)	Previous forensic history^c^ obtained via self-report.	Lifetime	Age at first offence: t = 2.27, p = 0.026 Age at first custody: t = 2.99, p = 0.004 Number of times in custody: t = -1.92, p = 0.059 Number of times on remand: t = -1.99, p = 0.053 Sentence length: t = -1.80, p = 0.075	23/42 Medium
Kubiak, 2004	Convenience	2004	Cross sectional	(M = 35.2, F = 38.1)	139/60	PTSD (current)	Recidivism^d^ measured using data obtained on prison/police databases following treatment program completion/release	Current	M: R^2^ = .06, p = 0.127 F: R^2^ = 0.02, p = 0.455	17/42 Low

^a^ Self Report Aggression Inventory (Little et al., 2003)

^b^ Self-Report Offending Scale (Huizinga et al., 1991)

^c^ Sentence length in months; Age of first offence; Age first time in custody; Number of times in custody; Number of times on remand

^d^ Defined as new arrest, parole revocation, or both

#### Aggressive behaviours

PTSD and violent or aggressive behaviour was assessed in nine studies using predominately male (n = 6) or adult samples (n = 5). Several (n = 6) studies reported positive associations between PTSD and aggression or violence, although few adjusted for potential confounders, and significant heterogeneity in measurement was also evident. Of note, one study of incarcerated youth reported that reactive, but not proactive, aggression was associated with PTSD symptoms [37]. Evidence supporting a relationship between PTSD and violent behaviour in adult prisoners was stronger among male compared to female samples, although one adult study which found significant associations between PTSD and aggression utilised a selected sample of male prisoners with comorbid substance use problems [53]. Five studies also examined the relationship between PTSD and self-reported anger or hostility, all of which found statistically significant associations (Table 3).

#### Offending behaviours

The role of PTSD in understanding offending and criminal behaviour was investigated in five studies, with limited evidence of an association (See Table 3). None were considered high quality. Studies were predominately comprised of adult (n = 4), male (n = 5) samples. Three studies explored links with reoffending, and three investigated the association between PTSD and the type and severity of prisoners’ offending.

## Discussion

### PTSD and comorbidity with other mental disorders

This systematic review of the association between PTSD and both other mental disorders and behavioural problems in youth and adult prison populations is based on 36 studies from 11 countries worldwide (Table 4). Results from meta-analyses indicated that the psychiatric disorder with the strongest association with PTSD was comorbid depression, followed by anxiety disorders. Prisoners with PTSD were also significantly more likely to have a substance use disorder, although the effect size was relatively small. A number of other disorders did not have sufficient data to permit a meta-analytic synthesis. Nevertheless, systematic review at least suggests the following. In adults, comorbidity with psychosis was less strongly evident than with neurotic disorders, and there was an association between PTSD and Cluster B personality disorders, particularly among female prisoners. A possible inverse relationship was observed between PTSD and ASPD with psychopathic features in men [55]. In adolescents, PTSD was not found to be any more likely to present among those with ADHD or conduct disorder than those without.

**Table 4:** Overview of associations with PTSD.

	Psychiatric Comorbidity											Problematic Behaviours					
Study	Psych Com	Aff Dis	Anx Dis	Psyc Dis	PD	Psych-pthy	CD	ADHD	CU traits	Subs Mis	Alc Mis	Suicid	Self Harm	Viol Aggres	Anger	Off. Behav.	Recidiv
Abram (2007)	+	0	+				0	0		+	+						
Ardino (2013)																	+
Ariga (2008)		0	+	+			0	0		0	0	0					
Becker and Kerig (2011)																+	
Caraballo (2013)		+	+									+					
Collins and Bailey (1990)														+			
Dixon (2005)	+	+	+	+			0	0		+		+					
Ford (2018)		+								0		+			+		
Giarrantano (2017)	+									+							
Gibson (1999)		+	+	0	+					0	0						
Gobin (2015)					0	0											
Hamerlynck (2008)														+			
Harner (2015)		+	+	0	+					0	0	+	+				
Heffernan (2015)	+	+	+	+						+	0	+					
Howard (2017a)												+	+				
Howard (2017b)										+				+			
Huang (2006)															+		
Karatzias (2017)																+	
Kerig (2016)		+															
Kerig (2009)		+								+		+			+		
Kimonis (2011)									0					0	+		
Kubiak (2004)																	0
McCallum (2018)														+			
Moore (2013)	+									0	0	+	+			0	0
Moore (2016)								0									
Perez-Pedrogo (2018)		+	+					+		+		0					
Plattner (2007)												+					
Sharf (2014)									+								
Shepherd (2018)												+					
Stimmel (2014)														+			
Ulzen (2003)		+	+				0	0		0	+						
Wahlstrom (2015)														+			
Warren (2009)					+					0	+			0	+		
Willemson (2012)						-											
Woodfield (2017)						+											
Zlotnick (1997)		+			+					+							

**+** positive association;—negative association; **0** no significant associations

Psych Com = Psychiatric Comorbidity (any); Aff Dis = Affective Disroder; Anx Dis = Anxiety Disorder; Psyc Dis = Psychotic Disorder; PD = Personality Disorder; Psych-pthy = Psychopathy; CD = Conduct Disorder; ADHD = Attention Deficit Hyperactivity Disorder; CU traits = Callous Unemotional Traits; Subs Mis = Substance misuse; Alc Mis = Alcohol misuse; Suicid = suicidality; Viol/Aggres = Violence or aggression; Off. Behav = Offending behaviour; Recidiv = Recidivism

### PTSD and associated behavioural problems

Evidence for an association between PTSD and behavioural problems in prison was mixed (Table 4). Results from meta-analyses indicated a significant association between PTSD and measures of suicidality, with risk estimates slightly higher among male prisoners. Associations with measures of aggression or offending behaviours did not permit meta-analytic syntheses. However, systematic review suggests that there are significant associations between PTSD and aggressive behaviours, particularly in adult samples. Consistent with findings on psychopathy [55, 56], there was some indication in the literature that PTSD was not associated with instrumental violence [37], which could suggest that aggressive behaviour in PTSD occurs primarily in the context of arousal and reaction to perceived threat, as opposed to callousness or lack of empathy. This review found limited evidence of an association between PTSD and offending type or recidivism.

### Youth vs adult samples

Results from meta-analyses suggested that adult samples reported stronger associations with depression and anxiety compared to youth samples. The association between PTSD and substance misuse was stronger amongst studies of incarcerated youth. Findings must be considered in light of previous findings that rates of reported trauma and PTSD may be higher in youth samples compared to adult samples [49], and that younger age has been cited as a risk factor for outcomes including institutional violence or self-harm [5, 62]. Interestingly, only one identified study compared samples of both youth and adult prisoners, and reported no significant interactions between age and PTSD in the prediction of anger and hostility [49].

### Impact of gender

Rates of PTSD in prison are higher amongst females compared to males [3]. However, our meta-analyses found stronger effect sizes among male samples for depression, anxiety and substance use comorbidities. This finding is consistent with a previous high quality study which highlighted that males with PTSD were more likely to have comorbid disorders compared to females with PTSD [28]. Gender differences in the types of mental disorders and behaviours examined by studies were also noted. ASPD, Psychopathy, ADHD and CU traits were more frequently investigated in male samples, while comorbidity between PTSD and BPD were only investigated in female studies. Similarly, problems relating to externalising behaviour (violence, aggression, offending) were investigated more amongst male prisoners, while internalising (suicidality, self-harm) behaviours were more consistently examined in female samples.

### Strengths and limitations

This is the first systematic review and meta-analysis, to our knowledge, to investigate associations between PTSD and comorbid mental disorders and problematic behaviours in prison populations. It included studies of both imprisoned youth and adults which employed validated tools to measure PTSD diagnosis and symptoms.

One of the main limitations of this review was the methodological heterogeneity between the studies, such as variations in the time period of measurement of both PTSD and comorbidities (i.e. past year or lifetime), varying definitions of outcome measures, and differences in the criminal justice characteristics of the sample (i.e. short-term detainees vs sentenced prisoners). Most of these studies were cross-sectional in design, limiting any causal inferences. Only four [28, 42, 46, 49] studies identified by this review were considered high quality, and many included studies had small sample sizes (<100). Of 36 studies examined, only 12 took account of potential confounders which may have explained any associations identified in simple group comparisons (or univariate analyses). Most domains explored using meta-analyses indicated significant heterogeneity. These variations made comparisons between studies challenging, precluding the use of meta-analyses in most cases, and meta-regression in all cases. It was also of note that only two studies identified by this review specifically investigated the construct of Complex PTSD (CPTSD) [32, 42]. In addition to symptoms of “simple” PTSD (i.e. re-experiencing, hypervigilance), CPTSD also requires disturbances in affect dysregulation, negative self-concept and interpersonal relationships [63, 64]. Findings from this review highlight the increasing need for research which differentiates between these two disorders, to examine their potentially distinct roles in adverse outcomes.

A final limitation was the lack of information on relationships with offending behaviour and recidivism. Limited to no relationship between PTSD and offending behaviour was identified by this review, however only five studies, the majority of which were cross-sectional, investigated such associations. Given that preventing recidivism remains a central task for those working in prisons, future research is needed to explore this further, and establish whether or not PTSD is prospectively linked with different forms of offending behaviour or criminal activity, as well as readmission to custody.

### Implications and conclusions

PTSD is a common disorder within prison populations [3]. People in prison are more likely to have experienced cumulative, multiple traumas across their lifetime [65], further increasing the risk of developing mental health problems [66], a pathway that may in part be mediated by the presence of PTSD symptoms [32, 61, 67]. The presence of PTSD has been linked to poorer treatment outcomes including functional impairment and treatment adherence [68]. However, PTSD often goes undetected by mental health services [69]. Screening for this disorder is not routinely embedded in clinical services, and the disorder typically remains un-diagnosed and untreated within prison settings [5, 7, 70]. The need for improved identification and treatment of PTSD in prison settings is further underscored by findings suggesting that spontaneous long-term remission rates of this disorder are modest [71], and that the evidence for the efficacy of short-term trauma-based therapies in this population is limited [72]. While the development of trauma-informed care is a welcome recent development, there is little consensus on how it is best defined or operationalized in prison settings [73, 74]. We have demonstrated that prisoners with PTSD are significantly more likely to also have comorbid depressive, anxiety or substance use disorder diagnoses, and that adult male prisoners with PTSD may be at the greatest risk of having co-occurring mental health difficulties. Findings suggesting associations between PTSD and suicidality also have important implications for future research into pathways to self-harming and suicidal behaviour in prison environments [5, 75]. While relationships with suicidal behaviour or ideation are likely to be complex and influenced by several factors, including comorbid disorders like depression, the specific role of PTSD has, until recently, been overlooked [5]. Therefore, improved screening and identification of PTSD is essential to improve access to clinical treatment and should be prioritised as an important first-step.

Finally, while this review found evidence for several cross-sectional associations between PTSD and other important mental health and behavioural problems, there was a notable lack of studies which investigated prospective outcomes–only two studies identified by this review employed a longitudinal design, both measuring readmission to custody. Thus, while there was evidence of associations between PTSD and suicidality or aggression, causal relationships between PTSD and subsequent risk of such adverse outcomes could not be assessed. This review has therefore highlighted the lack of robust research in this area and the need for future longitudinal studies utilising standardised and validated measures of both PTSD and outcomes, to explore the longer-term impact of PTSD on youth and adults in custody.

## Statements

The manuscript does not contain clinical studies or patient data. The authors declare that they have no conflict of interest.

## Supporting information

S1 Table Quality appraisal form. Quality appraisal form used to assess studies.
(DOCX)

S2 Table PRISMA checklist. Completed checklist of PRSIMA guidelines.
(DOC)

S1 Text Search strategy. Search strategy used in systematic review.
(DOCX)

S2 Text Prospero form. Form documenting registration with Prospero.
(PDF)
