# Supplementary material for: PTSD in prison settings: A systematic review and meta-analysis of comorbid mental disorders and problematic behaviours
Source: PLoS One. 2019 Sep 26;14(9):e0222407. doi: 10.1371/journal.pone.0222407 (PMC6762063; doi:10.1371/journal.pone.0222407)
Supplement: S1 Text — Search strategy used in systematic review. (DOCX) [file pone.0222407.s003.docx]

**S1 Text. Search Strategy.** Search strategy used in systematic review.

Search terms

Post-traumatic Stress Disorder terms used:

Posttraumatic Stress Disorder OR Post-traumatic Stress Disorder OR PTSD OR Posttraumatic Stress Reaction* OR Posttraumatic Stress Symptom* OR Post-traumatic Stress Symptom*

Prison terms used:

Prison* OR Incarcerate* OR Jail* OR Inmate OR Correctional Facility OR Correctional Institution OR Detain* OR Detention OR Penal Institution OR Penitentiar* OR Youth Offending Institution OR Young Offenders Institution OR Juvenile Detention

Combinations used

1. Exp Posttraumatic Stress Disorder/ or post-traumatic stress disorder

2. ("PTSD" or "posttraumatic stress disorder" or "post-traumatic stress disorder" or "posttraumatic stress" or "post-traumatic stress" or posttraumatic stress symptom* or post-traumatic stress symptom*).

3. Exp prisons 4. (prison* or incarcerate* or jail* or inmate or "correctional facility" or correctional institution or detain* or detention or "detention center" or penitentiar* or "penal institution" or “youth offending institution” or “young offenders institution” or “juvenile detention”)

5. 1 OR 2

6. 3 OR 4

7. 5 AND 6
