# Supplementary material for: PTSD in prison settings: A systematic review and meta-analysis of comorbid mental disorders and problematic behaviours
Source: PLoS One. 2019 Sep 26;14(9):e0222407. doi: 10.1371/journal.pone.0222407 (PMC6762063; doi:10.1371/journal.pone.0222407)
Supplement: S2 Text — Form documenting registration with Prospero. (PDF) [file pone.0222407.s004.pdf]

PROSPERO International prospective register of systematic reviews

Review title and timescale

1 Review title

Give the working title of the review. This must be in English. Ideally it should state succinctly the interventions or exposures being reviewed and the associated health or social problem being addressed in the review.

Prisoners with PTSD: A Systematic Review of Comorbidities and Outcomes

2 Original language title

For reviews in languages other than English, this field should be used to enter the title in the language of the review. This will be displayed together with the English language title.

3 Anticipated or actual start date

Give the date when the systematic review commenced, or is expected to commence.

06/03/2017

4 Anticipated completion date

Give the date by which the review is expected to be completed.

01/01/2018

5 Stage of review at time of this submission

Indicate the stage of progress of the review by ticking the relevant boxes. Reviews that have progressed beyond the point of completing data extraction at the time of initial registration are not eligible for inclusion in PROSPERO. This field should be updated when any amendments are made to a published record.

The review has not yet started ×

Review stage	Started	Completed
Preliminary searches	Yes	Yes
Piloting of the study selection process	Yes	Yes
Formal screening of search results against eligibility criteria	Yes	No
Data extraction	Yes	No
Risk of bias (quality) assessment	No	No
Data analysis	No	No

Provide any other relevant information about the stage of the review here.

Review team details

6 Named contact

The named contact acts as the guarantor for the accuracy of the information presented in the register record.

Emma Facer-Irwin

7 Named contact email

Enter the electronic mail address of the named contact.

emma.facer-irwin@kcl.ac.uk

8 Named contact address

Enter the full postal address for the named contact.

Institute of Psychiatry, Psychology and Neuroscience King's College London 16 De Crespigny Park SE5 8AF

9 Named contact phone number

Enter the telephone number for the named contact, including international dialing code.

07961854080

10 Organisational affiliation of the review

Full title of the organisational affiliations for this review, and website address if available. This field may be completed as 'None' if the review is not affiliated to any organisation.

Institute of Psychiatry, Psychology and Neuroscience

Website address:

11 Review team members and their organisational affiliations

Give the title, first name and last name of all members of the team working directly on the review. Give the organisational affiliations of each member of the review team.

Title	First name	Last name	Affiliation
Ms	Emma	Facer-Irwin	IoPPN
Dr	Deirdre	MacManus	IoPPN
Dr	Nigel	Blackwood	IoPPN

12 Funding sources/sponsors

Give details of the individuals, organizations, groups or other legal entities who take responsibility for initiating, managing, sponsoring and/or financing the review. Any unique identification numbers assigned to the review by the individuals or bodies listed should be included.

Institute of Psychiatry, Psychology and Neuroscience King's College London

13 Conflicts of interest

List any conditions that could lead to actual or perceived undue influence on judgements concerning the main topic investigated in the review.

Are there any actual or potential conflicts of interest?

None known

14 Collaborators

Give the name, affiliation and role of any individuals or organisations who are working on the review but who are not listed as review team members.

Title	First name	Last name	Organisation details

Review methods

15 Review question(s)

State the question(s) to be addressed / review objectives. Please complete a separate box for each question.

The objective of this review is to identify mental disorder comorbidity and behavioural outcomes associated with PTSD diagnoses and/or symptoms among male and female prisoners (both youth and adult offenders).

16 Searches

Give details of the sources to be searched, and any restrictions (e.g. language or publication period). The full search strategy is not required, but may be supplied as a link or attachment.

The following biomedical and social sciences databases will be searched: • EMBASE • MEDLINE • PsycINFO • Web of Science

17 URL to search strategy

If you have one, give the link to your search strategy here. Alternatively you can e-mail this to PROSPERO and we will store and link to it.

I give permission for this file to be made publicly available

Yes

18 Condition or domain being studied

Give a short description of the disease, condition or healthcare domain being studied. This could include health and wellbeing outcomes.

Posttraumatic Stress Disorder

19 Participants/population

Give summary criteria for the participants or populations being studied by the review. The preferred format includes details of both inclusion and exclusion criteria.

Samples include juvenile or adult offenders who were residing in prison (or youth-offending facility) at the time of assessment. Samples of prisoners of war will not be included. Offenders in other criminal justice settings (e.g.

probation, court) will not be included.

20 Intervention(s), exposure(s)

Give full and clear descriptions of the nature of the interventions or the exposures to be reviewed

This review will focus on factors associated with PTSD among youth and adult prisoners, with a specific focus on comorbid mental disorders and behavioural outcomes (e.g. offending, aggression, recidivism)

21 Comparator(s)/control

Where relevant, give details of the alternatives against which the main subject/topic of the review will be compared (e.g. another intervention or a non-exposed control group).

Male/female offenders Youth/adult offenders PTSD/no PTSD

22 Types of study to be included

Give details of the study designs to be included in the review. If there are no restrictions on the types of study design eligible for inclusion, this should be stated.

Included: Randomised controlled trials, non-randomised controlled trials, before and after studies, interrupted time series studies, parallel group studies, cohort studies, case-control studies, cross-sectional studies.

23 Context

Give summary details of the setting and other relevant characteristics which help define the inclusion or exclusion criteria.

Studies published in a book, theses/dissertations, conference papers, general comment papers, letters, editorials, reports, or other non-peer reviewed formats will be excluded.

24 Primary outcome(s)

Give the most important outcomes.

Offending behaviour outcomes (e.g. recidivism, violence, perpetration of offences) Comorbid mental disorders (e.g. Depression, Anxiety, Personality Disorder) Self-harm and Suicidal behaviour

Give information on timing and effect measures, as appropriate.

25 Secondary outcomes

List any additional outcomes that will be addressed. If there are no secondary outcomes enter None.

Any other identified correlates (e.g. anger, aggression, dissociation)

Give information on timing and effect measures, as appropriate.

26 Data extraction (selection and coding)

Give the procedure for selecting studies for the review and extracting data, including the number of researchers involved and how discrepancies will be resolved. List the data to be extracted.

Studies will be eligible for inclusion if they: (i) include male and/or female prisoners, including youth and adolescent in juvenile detention centres; (ii) identify PTSD using a validated diagnostic tool (e.g. the SCID) or a validated symptom checklist (e.g. the PCL-5) (iii) have been published between January 1990 and June 2017, (iv) present the results of peer-reviewed research based on intervention studies (e.g. randomised controlled trials, non-randomised controlled trials, parallel group studies), are before-and-after studies, interrupted time series studies, cohort studies, case-control studies, or cross-sectional studies; and (v) report on factors and/or outcomes associated with PTSD This review will accept multiple papers based on the same dataset if different data is reported in each paper which is relevant to this review. Data extraction: One reviewer will screen the downloaded titles and abstracts against the inclusion criteria; if it is unclear whether a reference meets the inclusion criteria, it will be taken forward to the next stage of screening. The reviewer will then assess the full texts of potentially eligible studies. Data from the included papers will be extracted onto standardised electronic forms by the reviewer. Extracted data will include information on study designs, sample characteristics and correlates of PTSD.

27 Risk of bias (quality) assessment

State whether and how risk of bias will be assessed, how the quality of individual studies will be assessed, and whether and how this will influence the planned synthesis.

Study quality will be independently appraised by two reviewers using criteria adapted from the Critical Appraisal Skills Program (CASP) checklists and using criteria adapted from validated tools. Inter-rater correlation will be calculated and disagreements will be resolved by discussion with a third reviewer before allocating a final appraisal score. The quality appraisal checklist will include items to assess study selection and measurement biases.

28 Strategy for data synthesis
Give the planned general approach to be used, for example whether the data to be used will be aggregate or at the level of individual participants, and whether a quantitative or narrative (descriptive) synthesis is planned. Where appropriate a brief outline of analytic approach should be given.
Odds ratios (ORs) and 95% confidence intervals (CIs) will be extracted or calculated for the association of correlates with PTSD among prisoners.

29 Analysis of subgroups or subsets
Give any planned exploration of subgroups or subsets within the review. 'None planned' is a valid response if no subgroup analyses are planned.
Analysis by age and gender is planned.

Review general information

30 Type and method of review
Select the type of review and the review method from the drop down list.
Systematic review

Crime and justice, Mental health and behavioural conditions

31 Language
Select the language(s) in which the review is being written and will be made available, from the drop down list. Use the control key to select more than one language.
English

Will a summary/abstract be made available in English?
Yes

32 Country
Select the country in which the review is being carried out from the drop down list. For multi-national collaborations select all the countries involved. Use the control key to select more than one country.
England

33 Other registration details
Give the name of any organisation where the systematic review title or protocol is registered together with any unique identification number assigned. If extracted data will be stored and made available through a repository such as the Systematic Review Data Repository (SRDR), details and a link should be included here.

34 Reference and/or URL for published protocol
Give the citation for the published protocol, if there is one.
Give the link to the published protocol, if there is one. This may be to an external site or to a protocol deposited with CRD in pdf format.

I give permission for this file to be made publicly available
Yes

35 Dissemination plans
Give brief details of plans for communicating essential messages from the review to the appropriate audiences.
Do you intend to publish the review on completion?
Yes

36 Keywords
Give words or phrases that best describe the review. (One word per box, create a new box for each term)
PTSD

Prison

37 Details of any existing review of the same topic by the same authors

Give details of earlier versions of the systematic review if an update of an existing review is being registered, including full bibliographic reference if possible.

38 Current review status

Review status should be updated when the review is completed and when it is published.

Ongoing

39 Any additional information

Provide any further information the review team consider relevant to the registration of the review.

40 Details of final report/publication(s)

This field should be left empty until details of the completed review are available.
Give the full citation for the final report or publication of the systematic review.
Give the URL where available.
